# Systematic Analysis of Main Constituents in Rat Biological Samples after Oral Administration of the Methanol Extract of Fructus Aurantii by HPLC-ESI-MS/MS

**Published:** 2014

**Authors:** Jingze Zhang, Wenyuan Gao, Zhen Liu, Zhidan Zhang, Changxiao Liu

**Affiliations:** a *Department of Pharmacy, Logistics College of Chinese People’s Armed Police Forces, Tianjin 300162, China.*; b *School of Pharmaceutical Science and Technology, Tianjin University, Tianjin 300072, China.*; c *The State Key Laboratories of Pharmacodynamics and Pharmacokinetics, Tianjin, 300193, China.*

**Keywords:** Fructus Aurantii, Metabolites, Plasma, Urine, Feces, HPLC-ESI-MS/MS

## Abstract

High performance liquid chromatography (HPLC) with diode array detection (DAD) and electrospray ionization tandem mass spectrometry (ESI/MS/MS) was used to analyze the main components in the methanol extract of Fructus Aurantii (FA) and the metabolites in rat biological samples after oral administration of the methanol extract of FA. There were 31 constituents identified in the extract of FA including 2 alkaloids, 1 coumarin, 10 flavonoid glycosides and 18 ploymethoxylated flavones. According to the UV spectrum and MS fragment character of main components in the methanol extract of FA, 18 parent constituents and 11 metabolites were tentatively identified in rat biological samples. Three groups of components in biological samples detected included flavonoid glycosides, their glucuronides and ploymethoxylated flavones. It was interested that flavonoid glycosides, their glucuronides and ploymethoxylated flavones can be investigated in rat plasma and urine, while in rat feces samples only flavonoid glycosides were detected. Triglycosyl, naringenin, neoeriocitrin, neoeriocitrin narirutin and hesperidin were the main components in rat feces which were found either in the plasma or in urine samples. However, naringin and neohesperidin were the main flavonoid glycosides which absorbed after oral administration. Except flavonoid glycosides and their glucuronides, ploymethoxylated flavones also the constituents absorbed because it was investigated mainly in rat plasma and urine but not in feces samples. The identification and elucidation of parent and metabolism components analyzed in biological samples provided the data for further pharmacological and clinical research on FA.

## Introduction

Fructus Aurantii (FA), one of the commonly used traditional Chinese medicines (TCMs), is derived from the dried and immature fruit of *Citrus aurantium* L. Citrus flavanone glycosides including naringin, hesperidin and neohesperidin (1) and plenty of ploymethoxylated flavones (2,3) had been separated and identified intensively during the past decades. Both flavanone glycosides and ploymethoxylated flavones were established to have a wide range of biological and physiological activities such as anti-inflammatory (3), anti-oxidant (4,5), anti-proliferative (6,7), anti-bacterial activities (8) and anticancer activity (9) and so on. Fructus Aurantii is an important component herb in traditional Chinese medicines such as Xue-Fu-Zhu-Yu Tang (10) and Wei-Chang-An Pill (11).

Plenty of investigations on FA ranged from chemical constituent, pharmacology, clinical treatment to pharmacokinetics. As for main constituents, numerous pharmacokinetic researches on flavonoids were performed for better understanding the course *in-vivo*. No report had displayed the main effective components systematically in rat biological samples (blood, bile, urine and feces) after oral administration of the extract of FA. 

As a powerful analytical tool, liquid chromatography coupled with DAD and electrospray ionization tandem mass spectrometry (LC/DAD/ESI/MS/MS) has been used to analyze the known constituents and elucidation of unknown ones in complex matrix (12,13). In our previous study, water extract of FA were dosed to rats, six parent components were detected in the plasma which identified flavonoids O-glycosides, and two metabolites were identified glucuronide (14). In this study, a systematical analysis of plasma, urine and feces was conducted for explaining the effective components* in-vivo* after multiple oral administration of the methanol extract of FA.

## Experimental

*Chemicals and materials*

HPLC grade acetonitrile was purchased from Fisher (USA). Water was purified by a Milli-Q water purification system (Millipore, USA). Methanol and acetic acid of analytical grade were purchased from Guangfu Technology Limited Company (Tianjin, China).

*FA* *preparation*

One hundred grams of FA was powdered and extracted with 1 L of methanol for 2 h in a reﬂux condenser. The ﬁltrate was collected and the residue was re-extracted twice. And then the solvent was removed under reduced pressure in a rotary evaporator (Buchi B-480). FA extract was gotten with a yield of 25.8%. The extract resolved in methanol and the final concentration is equal to 0.1 g crude drug/mL methanol. The methanol resolve centrifuged at 10,000 rmp for 10 min at 4 ℃ and the surpernatant was collected and filtered through a 0.22μm membrane for HPLC-ESI-MS/MS analysis. 

*Collection of biological samples*

Twelve wistar rats (♀), weighing about 280-320 g were used in the experiments. The animals were purchased from the Experimental Animal Center, Chinese Academy of Medical Sciences, Peking, SCXK-2007-004. The animals were housed in an environmentally (t=25 ˚C) and air humidity (60%) controlled room with a 12 h light-dark (07:00–19:00 h and 19:00–07:00 h) cycle, kept on a standard laboratory diet and drinking water ad libitum. All animals were fasted for 12 h with water ad libitum prior to the oral administration of the extract of FA. The methanol extract was made up to the concentration of 500 mg/mL in 0.5% carboxymethyl cellulose (CMC) suspension for rats’ oral administration.

Blank plasma, urine and feces samples were collected before oral administration of the extract of FA, then, each rat was administered orally doses of 8 mL/Kg FA extract every 1 h, after the third administration, blood samples were collected at 0.5, 1, 2, 3 h from the ophthalmic veins of the rats. The blood samples were centrifuged at 10,000 rmp for 10 min at 4 ℃ and the supernatants were decanted. 

Six male rats were first administered an oral dose of 8 mL/Kg of 0.5% carboxymethyl cellulose and held in metabolic cages for the collection of 2 h blank urine and feces samples. Then these rats were administrated orally to the extract at a dose of 8 mL/Kg. Urine and feces samples collected during 0-4 h after the third administration.

All samples were stored at -70 ℃ until analysis. This study was carried out in accordance with the “Regulation for the Administration of Affairs Concerning Experimental Animals (State Council of China, 1988).

*Extraction of rat biological samples*

The biological samples were treated with liquid–liquid extraction (LLE) which was added ethyl acetate. Eight hundred microliters of ethyl acetate was added to a clean tube containing a 200μL aliquot of the plasma, urine or fece sample (the fece sample was suspended in the water before LLE). After vortexing for 3 min, the samples were centrifugated at 10000 rpm for 10 min to obtain the supernatant. The supernatant was transferred to a fresh tube and dried with nitrogen at 45 ℃. The dried residue was dissolved in 100μL methanol. A 10μL aliquot of sample was analyzed by LC-MS.

*HPLC analysis*

All analyses were performed on an Agilent 1100 liquid chromatograph system (Agilent Technologies, USA), equipped with a quaternary pump, an online degasser and a column temperature controller, and coupled with a DAD as the detector. The analytical column was a Kromasil-C_18_ (250 mm × 4.6 mm *i.d*., 5 μm particle size). The column temperature was kept at 35 ℃. The mobile phase was a linear gradient prepared from acetonitrile (A), methanol (B), and 0.5 % ammonium acetate aqueous solution (C). The composition of the gradient was A-B-C, 4.3:0.7:95 at 0 min, 20:2.5:77.5 at 15 min, 22:3.5:74.5 at 40 min, 50:8:42 at 70 min, 69:11:20 at 100 min and then the system was returned to initial conditions. The flow rate was 0.8 mL/min, and the injection volume was 10 μL. UV absorbance was monitored at 230, 254, 280 nm using DAD. 

*HPLC- ESI-MS/MS analysis*

Samples were analyzed using an Agilent HPLC–MS system containing of a surveyor auto-sampling system (Agilent Technologies, USA), and an LC/MSD Trap XCT electrospray ion trap mass spectrometer. Source settings used for the ionization were: nebulizer gas flow, 70.00 *psi*; dry gas flow, 11.00 L min^-1^; electrospray voltage of the ion source, 3000 V; capillary temperature, 350 ℃; capillary exit, −158.5 V; skimmer, 40V. Nitrogen (>99.99%) and He (>99.99%) were utilized as sheath and lamping gas, respectively. The full scan of ions ranging from m/z 100 to 1200 in the positive and negative ion mode was carried out. The fragment ions were obtained using collision energy of 35% for both MS^2^ and MS^3^ experiments. Analyses were conducted at ambient temperature and the data were operated on the Xcalibur software.

## Results and Discussion

*Optimization of HPLC and MS conditions*

**Figure 1:**
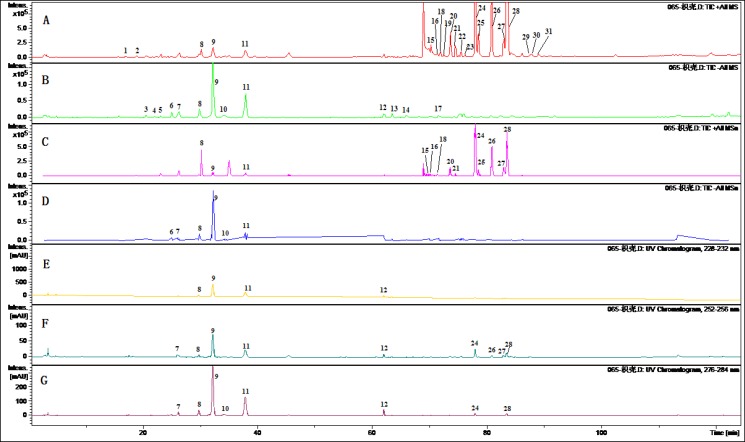
Chromatograms of the extract of Fructus Aurantii by LC-MS/MS. (A) TIC chromatogram in positive ESI mode.(B) TIC chromatogram in negative ESI mode.(C) TIC chromatogram in negative ESI mode of MS^n^.(D) TIC chromatogram in ESI positive mode of MS^n^. (E)HPLC chromatogram at 254 nm. (F) HPLC chromatogram at 230 nm. (G) HPLC chromatogram at 280 nm

To obtain chromatograms with good resolution of adjacent peaks, some HPLC analytical parameters including separation column, mobile phase and its elution mode were all investigated. Several trials were tried to achieve the good separation which included three kinds of C_18 _reversed-phase columns (Agilent ZOR-BAX, HiQ, Kromasil) and three gradient elution systems of methanol-water, acetonitrile-water and acetonitrile-methanol-water. The ratio of acetonitrile and methanol were selected at 50 to 8 and 0.5 % ammonium acetate aqueous solution were most suitable for the eluting solvent system as well as adequate for further MS/MS analysis in all the samples. MS parameters including spray voltage, capillary temperature, sheath gas and auxiliary gas pressure were optimized by analyzed the extract of FA. The optimized analysis methods to identify the chemical constituents in FA were developed by ESI-MS/MS in positive mode. In this study, chemical constituents in the extract of FA were scanned in positive and negative ion mode. As shown in Figure 1, it was found that the negative ion mode was more sensitive than the positive ion mode for flavonoid glycoside while the positive ion mode was more suitable for most polymethoxylated flavones.

*MS and UV analysis in the extract of FA*

In the analysis condition, the screening and further identification of main components in the extract of FA were conducted by HPLC-ESI-MS/MS in both the positive and negative ion modes and UV chromatograms at 230, 254 and 280 nm were set to gain the full chemical information. Thirty-one components were identified tentatively by comparing their UV spectrum, molecular weight and MS fragmentation behavior and the data were listed in Table 1. 

**Table 1:** Analysis of main components in the extract of Fructus Aurantii

**Peak**	**RT** **(min)**	**MS**		**MS/MS**		**Identification**
		**[M+H]** ^+^	**[M** **-** **H]** ^-^	**[M+H]** ^+^	**[M** **-** **H]** ^-^	
1	15.6	168	-	[168]: 124		Synephrine
2	18.9	152	-	[152]: 108		N-methyltyramine
3	20.5	-	741		[741]: 579, 433, 271	O-triglycosyl naringenin
4	21.9	-	741		[741]: 579, 433, 271	O-triglycosyl flavones
5	23.1	-	771		[771]: 609, 463, 301	O-triglycosyl hesperetin
6	25.0	-	595		[595]: 433, 287	Neoeriocitrin
7	26.2	-	595		[595]: 433, 287	Neoeriocitrin
8	29.8	-	579		[579]: 433, 417, 271	Narirutin
9	32.2	-	579		[579]: 433, 417, 271	Naringin
10	34.3	-	609		[609]: 463, 301	Hesperidin
11	37.8	-	609		[609]: 463, 301	Neohesperidin
12	61.9	-	593		[593]: 447, 431, 287	Poncirin
13	63.6	-	271		[271]: 256	Naringenin
14	65.9	-	301		[301]: 287	Hesperetin
15	70.1	392		[392]: 373		Trihydroxy-tetramethoxyflavone
16	71.2	389		[389]: 374		Monohydroxy-pentamethoxyﬂavone
17	71.5		227		-	Seselin
18	71.8	433		[433]: 418, 403		Heptamethoxyflavone
19	72.4	403		[403]: 388, 373		Hexamethoxyﬂavone
20	73.6	373		[373]: 358, 297		Pentamethoxyﬂavone
21	74.4	343		[343]: 313, 285		Tetramethoxyﬂavone
22	75.4	433		[433]: 418, 403		Heptamethoxyflavone
23	76.1	403		[403]: 388, 373		Hexamethoxyﬂavone
24	77.9	403		[403]: 388, 373		Hexamethoxyﬂavone
25	78.4	343		[343]: 328, 313		Tetramethoxyﬂavone
26	80.8	433		[433]: 428, 403		Heptamethoxyflavone
27	82.9	419		[419]: 404, 489		Monohydroxy-hexamethoxyflavone
28	83.4	373		[373]: 358, 297		Pentamethoxyﬂavone
29	87.4	389		[389]: 374, 259		Monohydroxy-pentamethoxyﬂavone
30	87.7	405		[405]: 390, 375		Hexamethoxyﬂavone
31	88.8	433		[433]: 428, 403		Heptamethoxyflavone

According to the literatures (15-18), the dominant fragmentation pathways of authentic compounds were studied. All authentic constituents displayed [M+H]^+^ in positive ion mode and [M-H]^- ^in negative mode. As for glycosides, there were two signals produced which corresponding to the pseudomolecular ions and its protonated aglycones in negative ion mode. In this study, *O*-triglycosyl and *O*-diglycosyl flavonoid glycosides were identified by analysis of the fragmentation pathways in MS/MS. The molecule ion at m/z 741, a *O*-triglycosyl flavonoid glycosides in negative ion mode showed MS^2^ fragment ion at m/z 579 due to the loss of the glucose reside, while *O*-diglycosyl flavonoid glycosides displayed MS^2^ fragment ion at m/z 417 due to the loss of glucose resides. Two *O*-triglycosyl naringenins, narirutin and naringin gave the major product ion at m/z 271 which was identified the protonated aglycones of these four components. The results were consistent with our previous study.

A plenty of ploymethoxylated flavones were investigated in this detected conditions. In positive mode, the pseudomolecular ions of ploymethoxylated flavones were more selective and more sensitive than in negative mode. According to their UV spectra and MS fragmentation characters (19-21), the pseudomolecular [M+H]^+^ ion at m/z at 373, 389, 359, 403 were tentatively identified as ploymethoxylated flavones. The MS^2^ fragment ion predominantly loss the different number of methyl radical and gave the ions at m/z [M-CH_3_+H]^ +^ or [M-2CH_3_+H]^ +^. Except the fragment of loss of methyl radical, m/z [M-CH_3_-H_2_O+H]^+^, [M-CH_3_-CO+H]^+^, [M-2CH_3_-H_2_O+H]^+^ and [M-2CH_3_-CO+H]^+ ^were detected in the literatures. In this study, 31 components were investigated in the methanol of FA. So the analysis methods were utilized for the further identification of the biological samples after oral admhinistration of the extract of Fructus Aurantii in rats.

*Analysis of the biological samples*

To elucidate the chemical constituents responsible for the pharmacological activity, it is essential to study the components profile *in-vivo* after administration (22, 23). The full scanning of constituents in rat plasma, urine and feced were analyzed by the same analysis method of the extract of FA. To avoid the interference of the endogenous, various sample preparation methods including protein precipitation with acetonitrile, methanol and liquid-liquid extraction were test to optimize an efficient clean up of the biological samples for obtaining better recovery of the target compounds. Finally, liquid-liquid extraction with ethyl acetate was chosen to prepare three kinds of the biological samples due to its simple, efficient and less interference from the endogenous matrixes.

**Figure 2:**
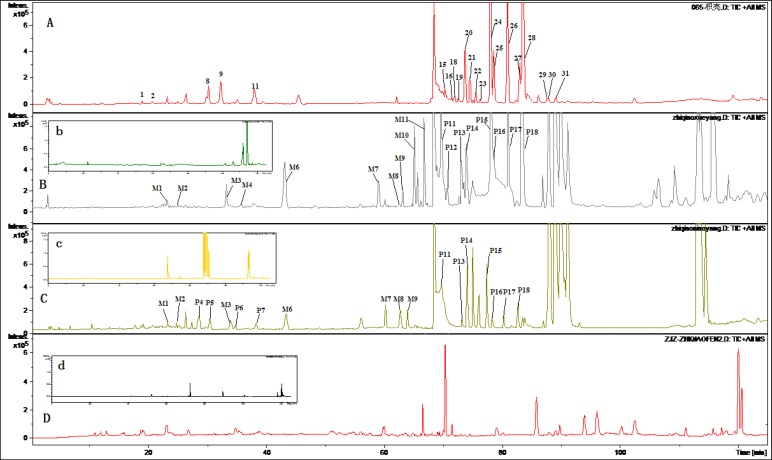
TIC Chromatograms of rat biological samples after oral administration of the extract of Fructus Aurantii in positive ESI mode. (A) TIC chromatogram of the extract of Fructus Aurantii; (B) TIC chromatogram of the rat plasma after oral administration (b: blank plasma); (C) TIC chromatogram of the rat urine after oral administration (c: blank urine); (D) TIC chromatogram of the rat faces after oral administration (c: blank feces).

**Figure 3:**
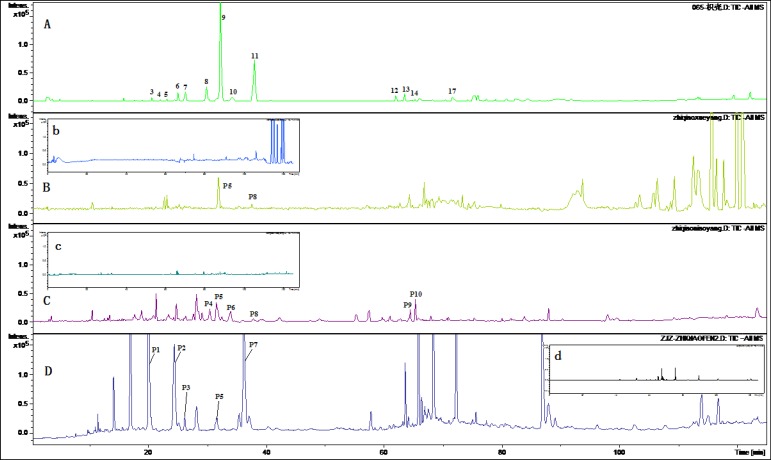
TIC Chromatograms of rat biological samples after oral administration of the extract of Fructus Aurantii in positive ESI mode. (A) TIC chromatogram of the extract of Fructus Aurantii; (B) TIC chromatogram of the rat plasma after oral administration (b: blank plasma); (C) TIC chromatogram of the rat urine after oral administration (c: blank urine); (D) TIC chromatogram of the rat faces after oral administration (c: blank feces).

Figures 2 and 3 displayed TIC chromatograms of the blank and drug-containing biological samples in positive and negative modes. By comparing the RT values, UV spectrum and MS/MS ion fragments characteristics of the peaks with the constituents detected in the extract of FA, the parent constituents were identified and some metabolites were observed in rat biological samples. Different kinds of components detected in biological samples were summarized in Tables 2 and 3. Compared with blank samples, 18 parent components and 11 potential metabolites were detected in the dosed rat biological samples. Although the parent and metabolites components in three kinds of biological samples were not consistent, it could be generally divided into three groups: flavonoid glycosides, their glucuronides and ploymethoxylated flavones.

As previous research shown that the major components detected in the plasma were flavonoid glycoside and their glucuronides (24), and polymethoxylated flavones (25) were also found absorbed* in-vivo*. In dosed plasma, only three flavonoid glycosides including naringin, neohesperidin, poncirin were found, while six flavonoid glycosides were detected in rats urine samples and six in the feces. These components were marked in Table 2. Triglycosyl naringenin was only found in feces but not in the plasma and urine samples. It can be concluded that this compound is not absorbed from intestinal tract. It is interested that ploymethoxylated flavones were detected in the plasma and urine samples. However, there were no ploymethoxylated flavones in rat feces. Therefore, they are completely absorbed. Tong *et al*. (26) investigated the pharmacokinetic of naringin, hesperidin, neohesperidin, naringenin and hesperetin in rat plasma after oral FA extract. The* C*_max_ and AUC of naringenin and hesperetin were higher significantly than that of flavanone glycosides. It is not glycoside but aglycone may play more important role *in-vivo*.

Eleven metabolites were identified by the approach shown in Figures 2 and 3. Five flavone glucuronides were detected as the metabolites of polymethoxylated ﬂavones. Except naringenin glucuronide and hesperetin glucuronide, which were detected consistence with the literatures, M3, M5 and M6 were identified as metabolites of polymethoxylated ﬂavones glucuronide. According to previous reports, the major metabolites of polymethoxylated ﬂavones were demethylated products of the parent components and their glucuronides (27). Components M7-M11 was identified as the demethylated products of polymethoxylated flavones. Except M4, M10 metabolites were detected in dosed plasma, while only four metabolites were investigated in the urine samples and no metabolites were detected in the feced.

**Table 2:** The parent components detected rat plasma (P), urine (U) and Feces (F) after oral administration of Fructus Aurantii

**Peak**	**Component ** **Name**	**TR** **(min)**	**MS**		**MS/MS**		**Identification**	**P**	**U**	**F**
			**[M+H]** ^+^	**[M** **-** **H]** ^-^	**[M+H]** ^+^	**[M** **-** **H]** ^-^				
**P1**	**3**	**20.5**		**741**		**[741]: 579, 433,271**	**O-triglycosyl naringenin**			**+**
**P2**	**6**	**25.0**		**595**		**[595]: 433, 287**	**Neoeriocitrin**			**+**
**P3**	**7**	**26.2**		**595**		**[595]: 433, 287**	**Neoeriocitrin**			**+**
**P4**	**8**	**29.8**	**581**	**579**		**[579]: 433, 417, 271**	**Narirutin**		**+**	
**P5**	**9**	**32.2**	**581**	**579**		**[579]: 433, 417, 271**	**Naringin**	**+**	**+**	**+**
**P6**	**10**	**34.3**		**609**		**[609]: 433,417,271**	**Hesperidin**		**+**	
**P7**	**11**	**37.8**		**609**		**[609]: 433,417,271**	**Poncirin**		**+**	**+**
**P8**	**12**	**6** **1** **.9**		**593**		**[593]: 447,431,287**	**Naringenin**	** +**	**+**	
**P9**	**13**	**63.6**		**271**		**[271]: 256**	**Neohesperidin**		**+**	
**P10**	**14**	**65.9**		**301**		**[301]: 287**	**Hesperetin**		**+**	
**P11**	**15**	**70.1**	**392**		**[** **392** **]:377, 362**		**Trihydroxy-tetramethoxyflavone**	** +**	**+**	
**P12**	**16**	**71.2**	**389**		**[** **389** **]** **:** **374, 359**		**Monohydroxy-p** **entamethoxyﬂavone**	** +**		
**P13**	**20**	**73.6**	**373**		**[** **373** **]:358, 343**		**Pentamethoxyﬂavone**	** +**	**+**	
**P14**	**21**	**74.4**	**343**		**[** **343** **]:328, 313**		**Tetramethoxyﬂavone**	** +**	**+**	
**P15**	**24**	**77.9**	**403**		**[** **403** **]:388, 373**		**Hexamethoxyﬂavone**	** +**	**+**	
**P16**	**25**	** 78.4**	**343**		**[** **343** **]:328, 313**		**Tetramethoxyﬂavone**	** +**	**+**	
**P17**	**26**	**80.8**	**432**		**[** **432** **]:417, 402**		**Heptamethoxyflavone**	** +**	**+**	
**P18**	**28**	**83.4**	**373**		**[** **373** **]:358, 343**		**Pentamethoxyﬂavone**	** +**	**+**	

**Table 3:** The metabolites components detected rat plasma (P), urine (U) and Faces (F) after oral administration of *Fructus Aurantii*

**Peak**	**TR** **(min)**	**[M+H]** ^+^		**P**	**U**	**F**
		**MS**	**MS/MS**			
M 1	22.5	449	[449]: 273, 153	+	+	
M 2	22.9	449	[449]: 273, 153	+	+	
M 3	29.4	535	[535]: 359, 328	+		
M 4	31.5	354			+	
M 5	31.9	565	[565]: 389, 359, 328	+		
M 6	43.1	581	[581]: 405, 390	+	+	
M 7	58.9	389	[389]: 359, 327	+		
M 8	62.4	329	[329]: 299	+		
M 9	63.0	419	[419]: 403, 389	+		
M 10	65.0	359	[359]: 329, 298	+		
M 11	65.5	329	[329]: 268	+		

**Figure 4:**
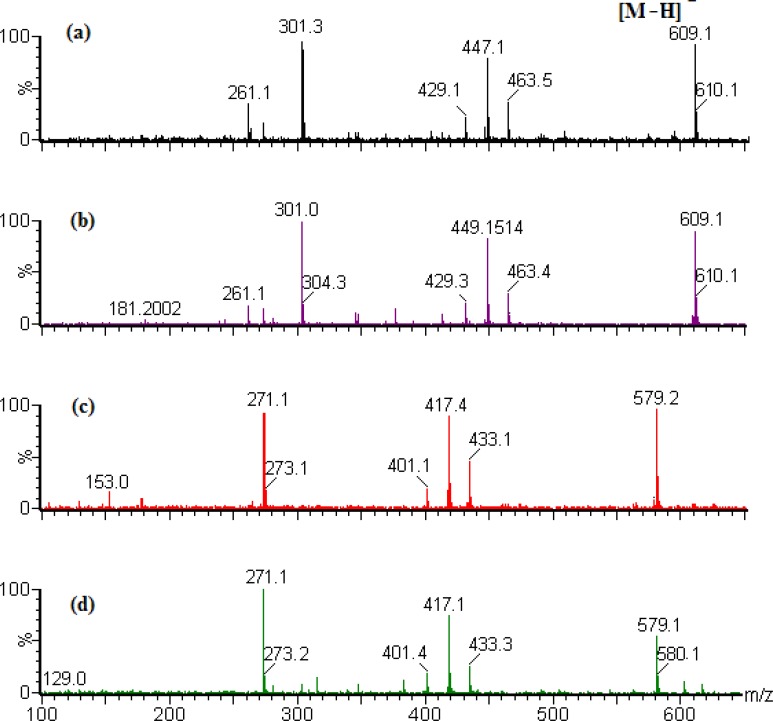
MS spectrum of the reference substances. (a) neohesperidin; (b) hesperidin; (c) naringin; (d) narirutin

**Figure 5:**
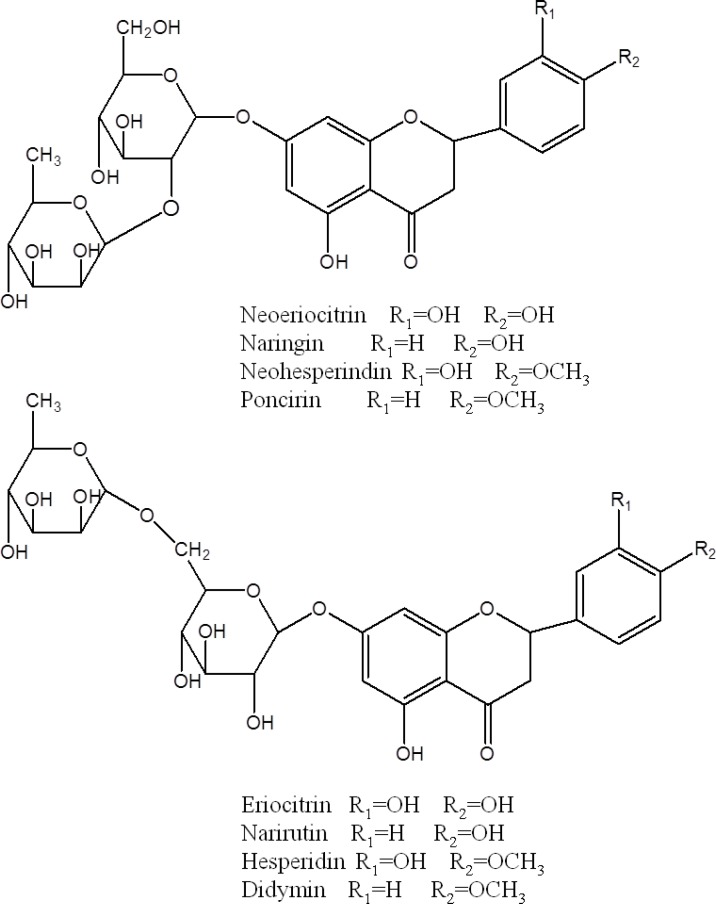
Structures of main identified compounds from the extracts of* F. aurantii*

## Conclusion

*In* *-* *vitro*, 31 constituents identified in the extract of FA including 2 alkaloids, 1 coumarin, 10 flavonoid glycosides and 18 ploymethoxylated flavones.* In-vivo, *18 parent components and 11 potential metabolites were detected in the dosed rat biological samples. 10 parent components and 10 potential metabolites were observed in plasma, while 14 parent components and 4 potential metabolites in urine. And in feces samples there were merely detected 5 parent components. Components M7-M11 as the demethylated products of polymethoxylated ﬂavones were newly detected in rat biological samples.
